# Multicellular crosstalk in neutrophilic asthma: unraveling the pathogenic web of steroid resistance and emerging precision therapies

**DOI:** 10.1186/s12967-026-08415-4

**Published:** 2026-06-13

**Authors:** Benhao Ye, Jialin Yao, Yuanyuan Wang, Ximing Liao, Di Wu, Muyun Wang, Xia Fang, Jing Gao, Qiang Li, Wei Gao

**Affiliations:** Department of Pulmonary and Critical Care Medicine, Shanghai East Hospital, School of Medicine, Tongji University, No. 150 Jimo Road, Pudong, Shanghai, 200092 P.R. China

**Keywords:** Neutrophilic asthma, Glucocorticoid resistance, Neutrophils, Cellular crosstalk, Precision medicine

## Abstract

**Background:**

Neutrophilic asthma (NA) is a severe, glucocorticoid-resistant phenotype. Although it represents a small proportion of asthma cases, it imposes a disproportionate burden due to its poor responsiveness to conventional therapies. Challenging the traditional view of isolated molecular defects, emerging evidence positions steroid resistance in NA as a product of dynamic, self-perpetuating pathogenic networks involving neutrophils, airway epithelial cells, T helper cells, macrophages, and B cells.

**Main body:**

This review synthesizes the cellular crosstalk underlying NA, emphasizing how convergent interactions impair glucocorticoid receptor function, a final common pathway to steroid resistance. We critically examine emerging therapeutic strategies that disrupt key network nodes, highlighting a shift from single‑target to rational combination regimens. In parallel, we propose a biomarker‑anchored endotyping framework for precision patient stratification, an essential step toward translating network‑targeted interventions into clinical practice. Finally, we identify major knowledge gaps in NA pathogenesis and steroid resistance, and outline priority directions for future research.

**Conclusions:**

This review provides an integrated perspective on the pathogenesis of steroid-refractory NA and offers actionable insights for precision management, aiming to improve clinical care for this severe asthma subtype.

## Introduction

Asthma is a chronic airway disease affecting approximately 260 million people globally, imposing a substantial burden of morbidity, mortality, and economic burden [1]. Its clinical heterogeneity is reflected in age of onset (childhood *versus* adulthood), inflammatory profiles (type 2-high *versus* type 2-low), and treatment responses. Although crude prevalence rates have plateaued in many regions since 2000, the absolute number of asthma cases continues to rise due to population growth, urbanization, and ageing demographics [2]. Within this growing disease burden, a significant subset of patients, particularly those with severe disease, responds poorly to current standard therapies.

Neutrophilic asthma (NA) is a heterogeneous type 2-low phenotype of severe asthma, characterized by persistent sputum neutrophilia, poor glucocorticoid responsiveness, late-onset disease, and frequent exacerbations triggered by infections or environmental irritants rather than allergens. It is often accompanied by comorbidities such as smoking and obesity. Diagnosis requires confirmation of severe asthma and exclusion of alternative conditions, with sputum neutrophil assessment as a key tool [3]. As a steroid-resistant phenotype, NA lacks a universal diagnostic threshold; studies vary in their cut-offs for sputum neutrophilia and handling of concomitant eosinophilic inflammation. Synthesizing evidence from studies that adopt a consensus-based definition (sputum neutrophils > 40%-76%, eosinophils < 2%) helps model the steroid-resistant core [4, 5]. Notably, different thresholds may select populations with varying inflammatory intensities, thereby influencing the reported dominance of specific immune pathways, a key consideration when comparing mechanistic studies.

Although NA accounts for only 16–22% of severe asthma cases [6], it imposes a disproportionate burden due to resistance to inhaled corticosteroids and biologics targeting Immunoglobulin (Ig) E (e.g., Omalizumab) or interleukin (IL)-5 signaling (e.g., Mepolizumab, Benralizumab) [2, 7]. Clinical cluster analysis indicates that, among non-type 2 asthma, NA is associated with greater disease severity and more frequent exacerbations [6]. Thus, research focused on this endotype is of high clinical relevance. Meanwhile, it is associated with elevated non-type 2 inflammatory mediators like IL-17, IL-6, and IL-8 [8]. A key distinguishing feature from classic eosinophilic asthma is its strong link to steroid resistance, posing a major therapeutic challenge [9]. Given the central role of corticosteroids, overcoming this resistance is critically important. Extensive research has focused on this asthma phenotype, yet many studies remain limited to isolated pathways or single targets, hindering a holistic understanding.

Previous review has highlighted core mechanisms of glucocorticoid resistance, including glucocorticoid receptor (GR) dysfunction (impaired nuclear translocation, reduced DNA‑binding affinity, and effects of respiratory infections) and decreased istone deacetylase 2 (HDAC2) activity [9]. NA is also strongly associated with T helper (Th) 17‑type inflammation, in which IL‑17 drives neutrophil recruitment [10]. However, clinical evidence consistently shows that blocking IL‑17 alone fails to alleviate symptoms or reverse steroid resistance [11]. Collectively, these findings indicate that Th17-driven neutrophilic inflammation represents a major, clinically challenging phenotype strongly linked to corticosteroid resistance [6, 12]. The limited efficacy of single-target therapies underscores the mechanistic complexity and shifts attention toward multifaceted cellular crosstalk. A central unresolved question is whether neutrophils actively perpetuate steroid resistance via specific intercellular interactions that sustain a pathological cycle. This review aims to comprehensively examine the dynamic crosstalk between neutrophils and key airway cells, mainly airway epithelial cells (AECs), T cells, macrophages, and plasma cells, and to evaluate how each axis contributes to the steroid-resistant inflammatory milieu. Deciphering these interactions is critical for developing rational, multi-targeted strategies to reverse steroid resistance in NA.

## The neutrophil-cell interaction network in NA

**Fig. 1:**
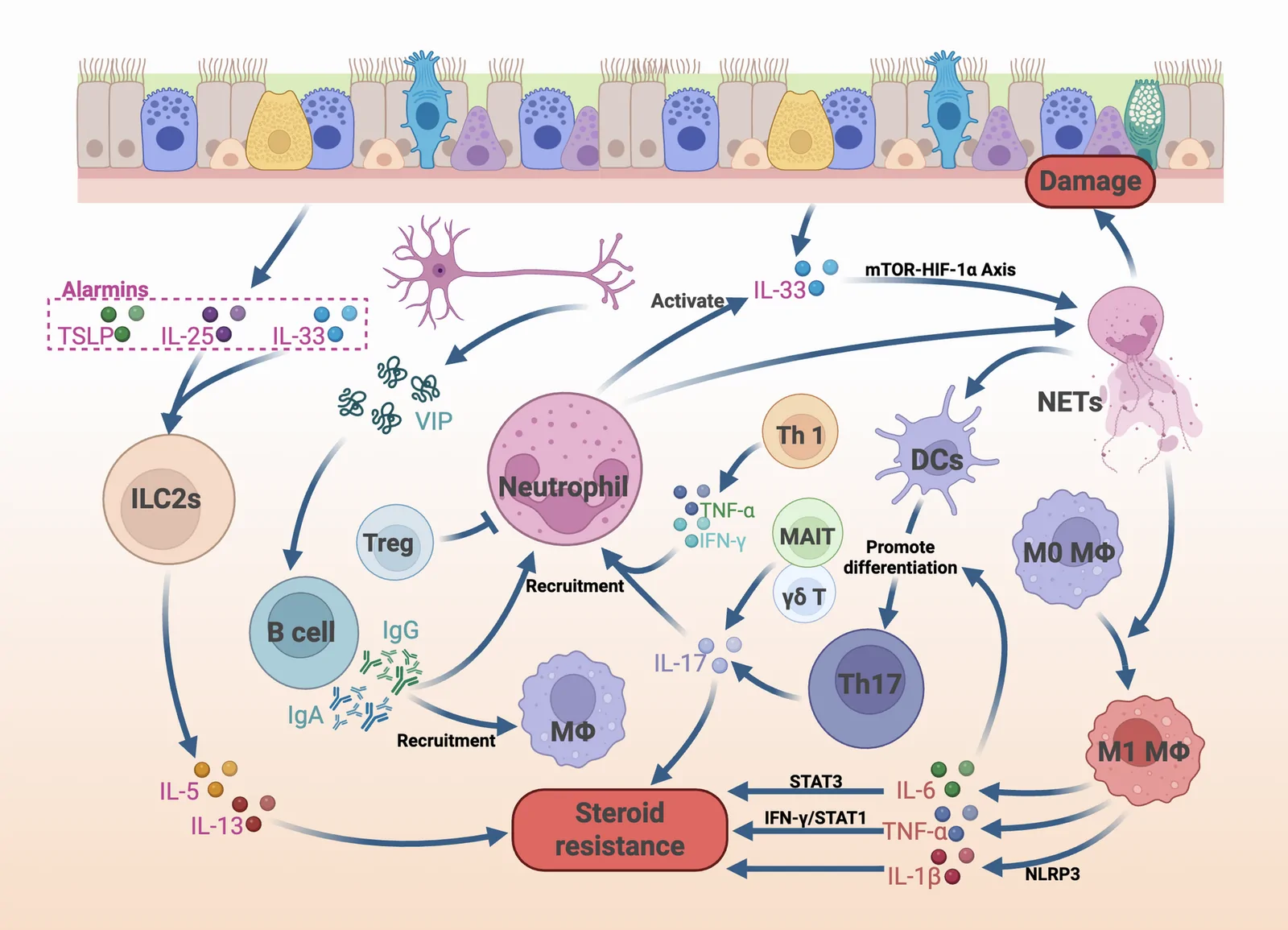
Neutrophil-centric cellular network orchestrating glucocorticoid resistance in NA. Abbreviations: DCs: dendritic cells; HIF: hypoxia-inducible factor; IFN: interferon; Ig: immunoglobulin; IL: interleukin; ILC2s: group 2 innate lymphoid cells; mTOR: mammalian target of rapamycin; MAIT: mucosal-associated invariant T cell; MΦ: macrophage; NETs: neutrophil extracellular traps; NLRP3: NOD-like receptor family, pyrin domain containing 3; STAT: signal transducer and activator of transcription; Th: T helper cell; TNF: tumor necrosis factor; Treg: regulatory T cell; TSLP: thymic stromal lymphopoietin; VIP: vasoactive intestinal peptide; γδ T: gamma delta T cell

Steroid‑resistant NA arises not from neutrophils in isolation but from a dysregulated multicellular airway ecosystem. Within this pathological network, neutrophils transition from terminal effectors to active inflammatory conductors, engaging in reciprocal crosstalk with other resident cells. Each interaction axis uniquely sustains a resilient inflammatory milieu and subverts glucocorticoid function. The neutrophil‑epithelial axis fuels a vicious cycle of barrier damage and pro‑inflammatory signaling. Neutrophils also promote a Th17‑polarized environment through T‑cell interactions and orchestrate a pro‑inflammatory, steroid‑resistant macrophage phenotype. Moreover, crosstalk with B cells triggers an autoantibody‑driven response that amplifies neutrophilic inflammation via complement activation. The following sections deconstruct these critical dialogues, analyzing how each axis collectively orchestrates the steroid‑resistant state in NA (Fig. 1).

### Neutrophil-AEC crosstalk

The airway epithelium constitutes the first line of respiratory defense. Upon inflammatory stimuli, it initiates the immune response by releasing chemokines such as IL-8, which recruit and activate neutrophils. Activated neutrophils then release neutrophil extracellular traps (NETs), the web-like structures composed of chromatin filaments and antimicrobial proteins. When exposed to AECs, NETs elicit a pro-inflammatory response characterized by IL-6 and IL-8 secretion and cause direct cellular injury, as indicated by the release of the cytotoxicity marker glucose-6-phosphate dehydrogenase [13]. Neutrophil elastase (NE) further damages AECs through direct proteolysis, fluid balance disruption, and inflammatory signal amplification [14]. This assault positions the neutrophil not merely as a terminal effector but as a primary instigator of dysregulated immunity.

The damage immediately triggers the epithelial alarm system, releasing alarmins including thymic stromal lymphopoietin (TSLP), IL-33, and IL-25. Neutrophils also activate these alarmins, for instance, by proteolytically processing the precursor form of IL-33 into its active form [15]. Thus, by causing initial damage and tuning alarm signals, neutrophils act as central catalysts in the inflammatory network. It is noteworthy that although clinical guidelines classify NA as a type 2‑low endotype, experimental models utilizing smoke exposure have shown upregulation of type 2 inflammatory mediators [16]. This observation, rather than representing a contradiction, highlights the potential contribution of type 2‑associated pathways, particularly those driven by group 2 innate lymphoid cells (ILC2s), to the pathogenesis of steroid‑resistant NA. Indeed, ILC2s are pivotal: IL‑25 promotes their proliferation, while IL‑33 directly activates them to produce substantial quantities of IL‑5 and IL‑13 [17, 18]. IL‑13 impairs glucocorticoid responses by suppressing T‑cell function and compromising epithelial innate immunity, thereby sustaining inflammation [19, 20]; it also upregulates 11β‑hydroxysteroid dehydrogenase type 2, a key glucocorticoid‑inactivating enzyme, in AECs and surrounding lung tissue [21]. Concurrently, IL-5 perpetuates a steroid-resistant eosinophilic inflammation driven by hormone-insensitive cells like ILC2s [22], which explains the efficacy of anti-IL-5 therapy in steroid-resistant patients [23]. More fundamentally, this activation generates a self-sustaining, cytokine‑rich milieu that impairs GR function through mechanisms such as reduced HDAC2 activity, directly linking the neutrophil-initiated cascade to steroid resistance. Additionally, IL-33-regulated autophagy depends on the mammalian target of rapamycin (mTOR)-hypoxia-inducible factor (HIF)-1α signaling axis, a central switch for NETs formation [16]. This creates a positive feedback loop that reinforces autophagy and NETs, underscoring the resilience of the neutrophil-centric network and its inherent resistance to monotherapies.

**Fig. 2:**
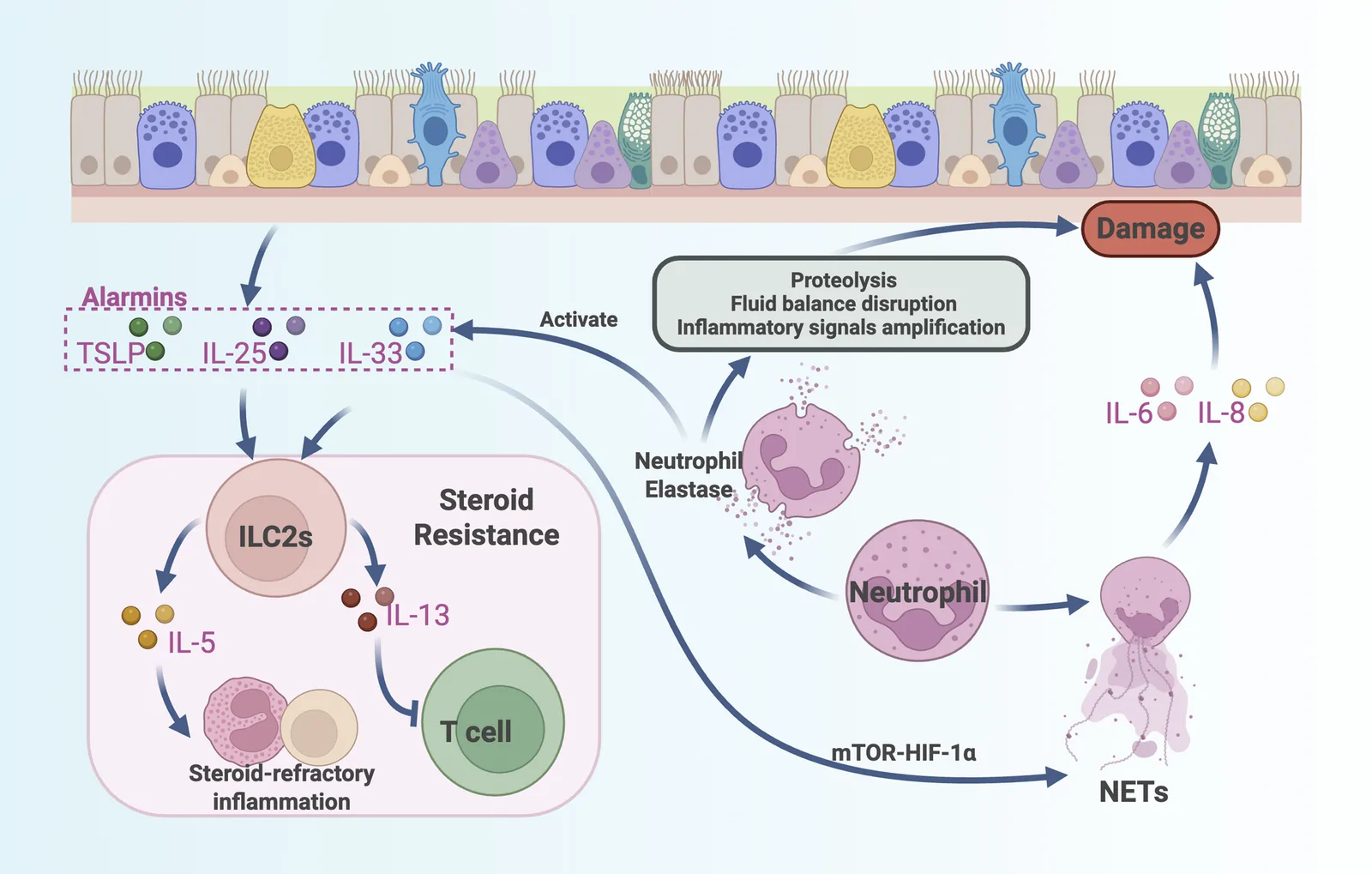
Neutrophil-epithelial cell crosstalk in glucocorticoid resistance. Abbreviations: HIF: hypoxia-inducible factor; IL: interleukin; ILC2s: group 2 innate lymphoid cells; mTOR: mammalian target of rapamycin; NETs: neutrophil extracellular traps; TSLP: thymic stromal lymphopoietin

In summary, the evidence delineates a pathogenic cascade: epithelial-derived signals recruit and activate neutrophils; neutrophil-derived NETs and NE then initiate epithelial damage; this damage, via alarmin-mediated activation of ILC2s, drives a steroid-resistant type 2 inflammation dominated by IL-13 and IL-5. This complex inflammatory milieu undermines corticosteroid efficacy. We hypothesize that the persistence of this microenvironment, rather than a static GR defect, is the primary driver of treatment failure. Future strategies to disrupt this network, for example, by combining alarmin inhibition with NE or NET-targeting agents, may be necessary to restore corticosteroid sensitivity (Fig. 2).

### Neutrophil-T cell crosstalk

Neutrophils and Th17 cells form a critical pathogenic axis in NA, driving persistent inflammation and corticosteroid resistance. The interaction begins with neutrophils: in severe asthma patients, neutrophil cytoplasts, enucleated bodies formed via NETs formation, are found in bronchoalveolar lavage fluid, and their abundance correlates with IL-17 levels [24]. Experimentally, these cytoplasts activate dendritic cells to drive Th17 differentiation [24, 25], positioning neutrophils as initiators of adaptive immunity. Th17 cells are not terminally differentiated; they exhibit plasticity shaped by the tissue microenvironment, cytokine signals, and metabolic state [26]. This plasticity allows their function to be modulated by the inflammatory milieu, which is heavily influenced by neutrophil activity. Through their hallmark cytokine IL-17, Th17 cells mediate neutrophil recruitment, antimicrobial peptide production, and mucosal barrier enhancement, striking a balance between defense and immunopathology [26].

A specific mechanism underpins Th17-mediated steroid resistance. Stress-induced glucocorticoids suppress the transcription factor T cell factor 1 while enhancing the signal transducer and activator of tanscription (STAT) 3/HIF-1α pathway in Th17 cells [27]. This metabolic reprogramming boosts glycolysis, proliferation, differentiation, and survival of Th17 cells, driving massive IL-17 production and subsequent neutrophil infiltration. We further hypothesize that NETs may amplify this process. Ultimately, this selective enhancement of Th17 activity counteracts the broad suppressive effects of glucocorticoids, thereby establishing a distinct cell-intrinsic mechanism of corticosteroid resistance. The transcription factor T-bet serves as a pivotal regulator, promoting Th1 development while inhibiting Th2 (via GATA binding protein 3) and Th17 (via retinoic acid receptor-related orphan receptor gamma t) pathways [28, 29]. T-bet deficiency leads to uncontrolled Th17 differentiation and excessive IL-17 production, driving neutrophil recruitment via chemokines like C-X-C Motif Chemokine Ligand 1/ C-X-C Motif Chemokine Ligand 8 and granulocyte colony-stimulating factor (G-CSF). This phenotype is validated in asthma models and reversible with anti-IL-17 treatment [30–32]. The neutrophil-rich microenvironment may further suppress T-bet, creating a network loop where neutrophils shape a T-cell landscape conducive to their own survival. Beyond intrinsic regulation, an extrinsic pathway also confers steroid resistance. In other contexts, myeloid‑derived IL-1β binds to the IL-1 receptor on Th17 cells, activating the myeloid differentiation primary response protein 88- interleukin-1 receptor-associated kinase 2-janus kinase2-STAT5 axis. Activated STAT5 then interacts with GR, reprogramming transcription to sustain inflammatory factors (e.g., IL-17 A, granulocyte-macrophage colony-stimulating factor) while suppressing anti-inflammatory genes (e.g., Gilz), rendering cells resistant to dexamethasone [33]. In the asthmatic airway, neutrophils and macrophages are plausible sources of IL‑1β, directly imposing glucocorticoid resistance on Th17 cells via this conserved pathway.

**Fig. 3:**
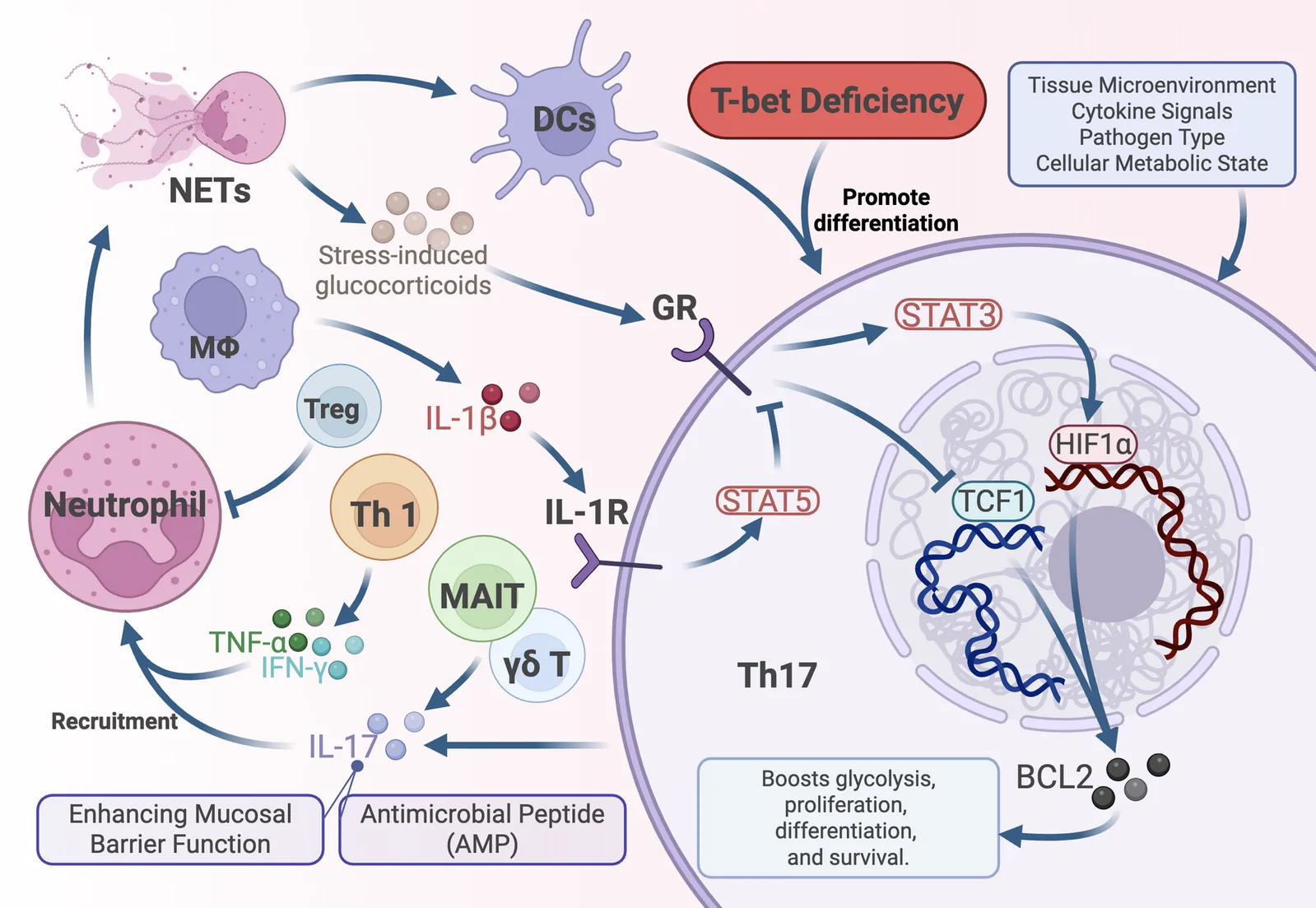
Neutrophil-T cell crosstalk in glucocorticoid resistance. Abbreviations: BCL2: B-cell lymphoma 2; DCs: dendritic cells; GR: glucocorticoid receptor; HIF: hypoxia-inducible factor; IL: interleukin; IFN: interferon; MAIT: mucosal-associated invariant T cell; MΦ: macrophage; NETs: neutrophil extracellular traps; STAT: signal transducer and activator of transcription; T-bet: T-box expressed in T cells; TCF1: T cell factor 1; Th: T helper cell; TNF: tumor necrosis factor; Treg: regulatory T cell; γδ T: gamma delta T cell

Taken together, this crosstalk reveals a pathogenic positive feedback loop: neutrophils initiate and amplify a Th17 response; Th17 cells, through intrinsic metabolic reprogramming and extrinsic IL‑1β/STAT5 signaling, adopt a steroid‑resistant phenotype; this in turn drives further IL‑17‑mediated neutrophil recruitment. This self-reinforcing network dynamically undermines GR function, making steroid resistance a consequence of sustained cellular crosstalk rather than a static defect. Beyond the Th17‑centric axis, other T cell subsets also contribute meaningfully. Mucosal‑associated invariant T cells and γδ T cells, for example, serve as alternative sources of IL‑17, amplifying neutrophil recruitment and activation [26, 34]. Severe NA often presents with a Th1/Th2 imbalance, and impaired regulatory T cell function may fail to restrain neutrophilic inflammation [34, 35]. Th1-associated cytokines, particularly IFN-γ and tumor necrosis factor-alpha (TNF-α), are elevated in severe, steroid-resistant asthma and participate in neutrophil recruitment and activation [36]. Thus, while the Th17‑neutrophil circuit remains the most potent driver, the broader T cell landscape in NA involves multiple interconnected subsets that collectively sustain the steroid‑resistant state (Fig. 3).

### Neutrophil-macrophage crosstalk

Macrophage phenotypic plasticity allows these cells to adapt dynamically to microenvironmental cues. In NA, neutrophils emerge as master regulators of this plasticity. Emerging evidence indicate that NETs serve as pivotal programming signals for macrophages, promoting a pro-inflammatory M1-like shift through direct interactions or microRNA transfer [37, 38]. Neutrophil-derived reactive oxygen species (ROS) also induce M1 polarization, amplifying inflammatory cascades [39]. Thus, neutrophils directly instruct macrophage polarization, positioning them as upstream architects of the immune response, a process classically attributed to stimuli like interferon-gamma (IFN-γ) and Toll-like receptor (TLR) ligands [40].

Once polarized, M1 macrophage sustain inflammation and steroid resistance, primarily by secreting cytokines, including TNF-α and IL-1β [41, 42]. IL-1β, often released upon NOD-like receptor family, pyrin domain containing 3 (NLRP3) inflammasome activation, amplifies a feed-forward loop that promotes neutrophilic inflammation and airway hyperresponsiveness, effectively blunting glucocorticoid responses [43]. IL-6 drives resistance via persistent STAT3 activation, impairing GR function by reducing phosphorylated GR and HDAC2 levels and suppressing the negative regulator Suppressor of Cytokine Signaling 3. IL‑6 also promotes Th17 differentiation and IL‑17 A release, exacerbating neutrophilic inflammation [44]. Beyond impairing local GR signaling, IL-6 facilitates neutrophil mobilization from bone marrow to lungs via C-X3-C motif chemokine receptor 1, ensuring a continuous supply of effector cells [45]. TNF‑α, synergizing with the IFN‑γ/STAT1 pathway, sustains nuclear factor kappa-light-chain-enhancer of activated B cells (NF-κB) activation, overriding glucocorticoid suppression even with an intact GR [46]. Furthermore, TNF-α synergizes with IL-17 A to stimulate G-CSF production, a key driver of NA [47, 48], reinforcing neutrophil dominance and creating a cytokine milieu that overwhelms glucocorticoid anti-inflammatory capacity.

**Fig. 4:**
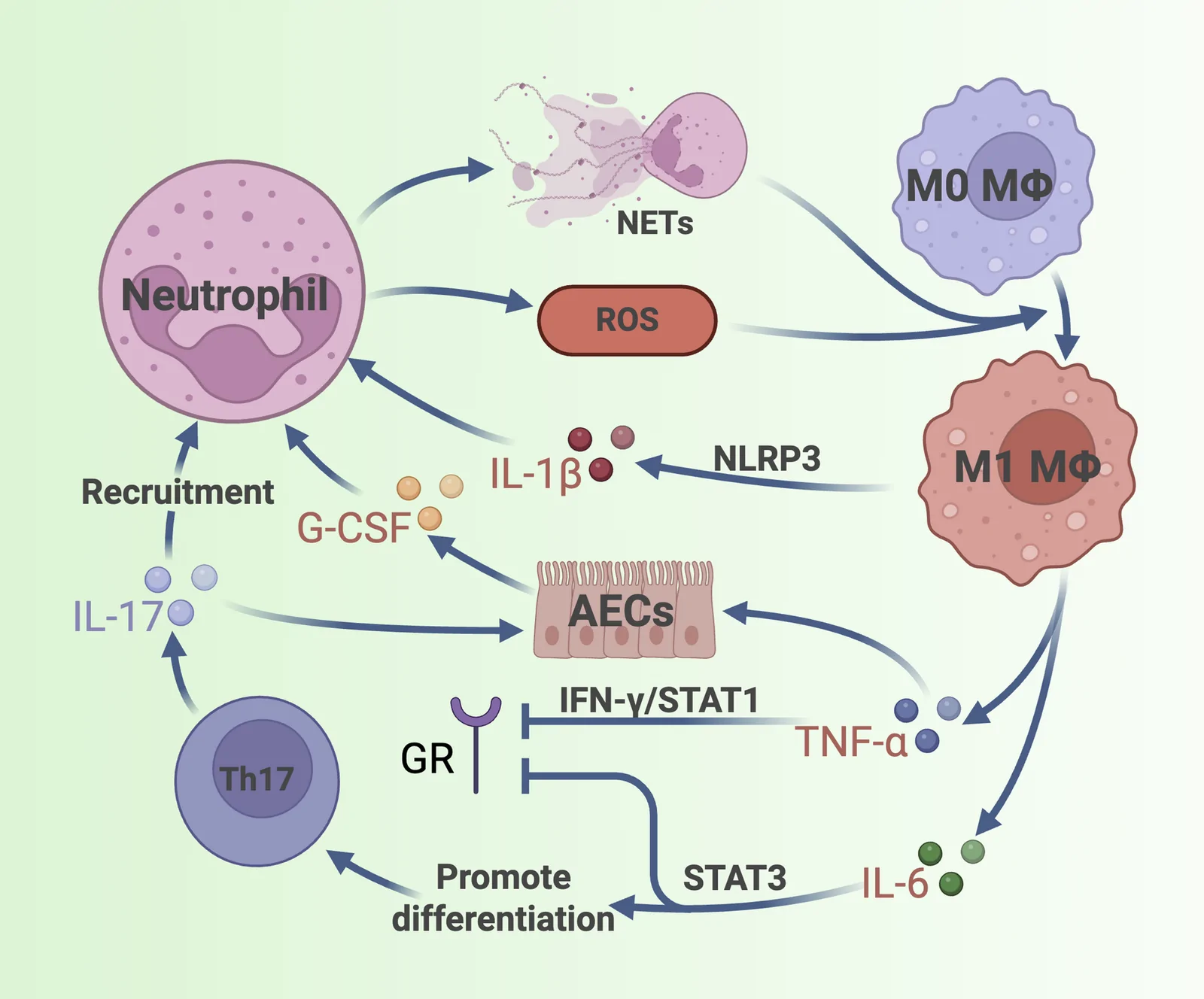
Neutrophil-macrophage crosstalk in glucocorticoid resistance. Abbreviations: G-CSF: granulocyte colony-stimulating factor; GR: glucocorticoid receptor; IFN: interferon; IL: interleukin; MΦ: macrophage; NETs: neutrophil extracellular traps; NLRP3: NOD-like receptor family, pyrin domain containing 3; ROS: reactive oxygen species; STAT: signal transducer and activator of transcription; TNF: tumor necrosis factor; AECs: airway epithelial cells

Collectively, these findings delineate a coherent pathogenic cascade: neutrophil-derived signals polarize macrophages toward an M1 phenotype, whose secretory products (TNF-α, IL-1β, and IL-6) orchestrate steroid resistance through convergent pathways. These effector molecules also fuel neutrophil recruitment and activation, creating a self-sustaining cycle of refractory inflammation. This cycle epitomizes steroid resistance as a network-driven phenomenon, initiated and sustained by neutrophil-macrophage crosstalk, which generates an inflammatory microenvironment inherently resistant to standard glucocorticoid therapy (Fig. 4).

### Neutrophil-B cell crosstalk

B cells play divergent roles across asthma endotypes. In allergic (type 2-high) asthma, they are responsible for producing allergen-specific IgE antibodies, which initiate downstream inflammatory cascades [49]. Conversely, in type 2-low NA, their pathogenic role shifts toward local production of elevated IgG and IgA [50]. This fundamental shift in B-cell function redefines their role from effectors of allergy to instigators of a separate, often steroid-resistant, inflammatory circuit in NA. Mechanistically, it points to the emergence of a localized autoimmune process, where autoantibody production catalyzes disease progression.

Local IgG/IgA production may facilitate the formation of pathological immune complexes within the airways. Upon tissue deposition, these complexes continuously activate the classical complement pathway, generating potent anaphylatoxins complement component 3a (C3a) and C5a [51]. This pathway could establish an innate immune activation loop that, once initiated by adaptive B-cell responses, proceeds independently of T-cell help and thus might evade certain immunoregulatory mechanisms. The anaphylatoxins C3a and C5a promote neutrophil and macrophage recruitment and activation via chemotaxis and specific receptor engagement [52]. In this context, B cells may act as instigators that leverage the complement system to mobilize neutrophils. This process could contribute to a self-sustaining cycle of neutrophilic inflammation, tissue damage, and pro-inflammatory cytokine release (e.g., IL-8, IL-1β). Notably, this pathway operates independently of Th2 cytokines, and the resulting neutrophil-driven inflammation is frequently associated with relative corticosteroid resistance. We further propose a testable hypothesis that the C3a/C5a-rich microenvironment leads to glucocorticoid resistance by directly impairing GR function, for instance through modulation of GR phosphorylation or co-repressor activity, which results in a functionally “weakened” GR state. This hypothesis provides a mechanistic direction for future investigation into steroid resistance in this asthma phenotype.

Clinical observations highlight this B cell-driven pathology. A separate study reported that, even after adjusting for oral corticosteroid use, the association between IgA^+^ memory B cells and small airway dysfunction remained significant [53]. This observation raises a critical possibility: in patients with mild-to-moderate asthma, a non-eosinophilic, B cell-associated process may contribute to a corticosteroid-insensitive phenotype. The findings imply that this B cell pathology may independently and substantially contribute to morbidity, and could represent a key determinant of steroid resistance even in less severe cases. Emerging evidence introduces a novel neuro-immune dimension. Neuro-immune interactions, such as the release of vasoactive intestinal peptide (VIP) by sensory neurons, can directly promote antibody production by B cells [54]. This introduces a fascinating dimension of neuro-immune crosstalk into the pathophysiology of NA. It suggests that neural signals, potentially elicited by tissue damage or environmental irritants, can directly stimulate B cells, thereby amplifying the local autoimmune component of the disease. Within this model, the resulting antibodies may not only drive pathology but could also opsonize bacteria, enhancing neutrophil-mediated phagocytosis and bactericidal activity.

In perspective, the VIP-B cell axis introduces a candidate target for intervention. Disrupting this neuro‑immune dialogue could selectively dampen local autoantibody production while sparing broader immunity. Additionally, the sustained presence of IgA^+^ memory B cells in airways raises the possibility of a persistent local immune memory, potentially sustaining steroid resistance and disease relapse. Overall, the coordinated activity of neural signals, B cells, and neutrophils supports a model in which these cells operate as an integrated pathogenic network. Here, neutrophils may also play a modulatory role, via feedback signals that perpetuate inflammation. Moving forward, this network‑based viewpoints to the importance of identifying and targeting critical intercellular communication hubs as a potential strategy for achieving therapeutic breakthroughs in steroid‑resistant asthma.

### Common hubs in cellular crosstalk

Owing to the predominant reliance on human cross-sectional studies and reductionist preclinical models in NA research, the in vivo regulatory mechanisms underlying multicellular crosstalk remain largely unclear. Nevertheless, several common hub components, including specific cellular players, cytokines, and signaling pathways, can be identified from the neutrophil‑mediated intercellular interactions described above. As web‑like structures generated upon neutrophil inflammatory activation, NETs provide a physical scaffold that supports sustained inflammatory insults and facilitates intercellular signal propagation. Through direct contact and via their scaffold‑associated NE and microRNAs, NETs induce inflammatory injury in AECs and activate Th17 cells and macrophages. Indeed, NETs serve as central mediators in neutrophil‑associated inflammatory disorders. Further research is needed to elucidate their spatiotemporal regulatory dynamics in NA, for which existing spatial transcriptomic frameworks from allergic asthma can serve as a reference [55, 56].

Th17 cells regulate adaptive immunity, neutrophilic inflammation and tissue immune homeostasis, playing critical roles in autoimmune disorders, chronic inflammatory conditions, and severe steroid-resistant respiratory diseases. In NA, Th17 cells are stimulated and polarized by neutrophil cytoplasts, NETs, IL-1β and epithelial alarmins, which activate the STAT3, HIF-1α and STAT5 pathways, inducing metabolic reprogramming and glucocorticoid resistance. Activated Th17 cells secrete IL-17 A and IL-17 F, which in turn induce AECs to secrete neutrophil chemoattractants and synergize with M1 macrophage-derived TNF-α to enhance G-CSF production. These form a self-sustaining loop driving continuous neutrophil infiltration and steroid resistance; however, the spatial targeting strategies and temporal regulation of Th17‑directed therapies require further investigation.

In NA, key molecules and signaling axes governing multicellular crosstalk include STAT, NF-κB, the NLRP3 inflammasome, the mTOR-HIF-1α axis, and the GR. Through convergent intercellular crosstalk, these regulators mediate inflammatory amplification, macrophage polarization, epithelial injury, and glucocorticoid resistance. Targeting these core molecules can disrupt the self‑sustaining inflammatory network, restore GR function, and reverse steroid resistance, thereby offering crucial precision therapeutic targets. Collectively, these common hubs of multicellular crosstalk in NA represent valuable targets for reversing glucocorticoid resistance. They directly or indirectly control multiple pathways leading to steroid resistance, and their targeted modulation may therefore yield superior therapeutic outcomes. Nevertheless, the design of targeted regulatory strategies depends on elucidating the spatial relationships and temporal features of these common hubs, an endeavor that can be advanced through spatial transcriptomics, organoid systems, and three‑dimensional co‑culture models.

## Targeting the neutrophilic network to overcome steroid resistance

Despite corticosteroids remaining the cornerstone of asthma management, many patients with neutrophilic inflammation respond poorly. This steroid resistance is not a single defect but the pathological endpoint of the multicellular network described above. At its molecular core lies a final common pathway: impaired GR function, through reduced expression, altered phosphorylation, disrupted nuclear translocation, or aberrant epigenetic modifications. Translating this understanding into practice requires moving beyond broad immunomodulation. The next frontier is developing precision combination therapies that concurrently target key nodes within the neutrophilic inflammatory circuit, thereby resensitizing the airway to corticosteroids. The following sections explore such strategies, aligned with the major cellular axes driving disease.

### Convergent regulation of GR dysfunction

Glucocorticoid resistance in NA arises from a self-amplifying inflammatory network, with impaired GR function as its convergent endpoint. This pathogenic cascade begins with neutrophil-derived products (NETs, NE) that damage the airway epithelium, triggering alarmin (TSLP, IL-33, IL-25) release. These alarmins activate ILC2s to drive steroid-resistant type 2 inflammation via IL-13 and IL-5. Concurrently, a positive feedback loop between neutrophils and Th17 cells is reinforced by glucocorticoid-driven metabolic reprogramming via STAT3/HIF-1α pathway and inflammatory signals like IL-1β/STAT5 axis. Within such milieus, some Th17 subsets may acquire a treatment-resistant phenotype. For example, multidrug resistance protein 1 positive Th17.1 cells resist steroids through combined efflux-independent functions and pro-inflammatory transcriptional programs [57]. Neutrophils also polarize macrophages toward an M1 phenotype, which secretes TNF-α, IL-1β, and IL-6, sustaining activation of NF-κB, the NLRP3 inflammasome, and STAT3 signaling. An additional autoimmune layer, B cell-derived IgG/IgA immune complexes and complement activation (C3a/C5a), perpetuates neutrophilic inflammation. Critically, these distinct pathways converge to impair GR function through multiple synergistic mechanisms.

GR impairment, the final common pathway of treatment failure, can result from intrinsic genetic/epigenetic defects or extrinsic inflammatory reprogramming. Intrinsic alterations include imbalances in GR splice isoform expression (e.g., increased GRβ or altered GRα/GRβ ratio) [58–60], GR polymorphisms (single nucleotide polymorphisms) [61], abnormal post-translational modifications, chaperone dysfunction, and altered membrane GR signaling (involving GRγ) [58]. A key example is the ER22/23EK polymorphism in the transactivation domain (exon 2), which changes two adjacent codons from glutamic acid-arginine to glutamic acid-lysine [62]. This single nucleotide polymorphism reduces GR‑mediated transactivation without affecting NF‑κB transrepression, thereby altering the glucocorticoid sensitivity [62, 63]. In NA, however, the inflammatory milieu plays a dominant role in disabling GR. IL-13 impedes GR nuclear translocation, IL-6 disrupts GR phosphorylation, and TNF-α-sustained NF-κB activation hinders GR-DNA binding. Most critically, this microenvironment induces epigenetic alterations, primarily by reducing HDAC2 activity/expression via oxidative stress and cytokine signaling, crippling the GR’s anti-inflammatory transcriptional efficacy and cementing resistance [58] (Table 1).

**Table 1:** Mechanisms of GR dysfunction in NA

Stage	Responsible Cells	Mediators	Mechanisms	References
Pre-receptor	MDR1^+^ Th17 cells	IL-17, IL-22, IFN-γ	Mediated by drug efflux and intrinsic pro‑inflammatory transcription	Ramesh, et al. 2014
	AECs	11β‑HSD2	Convert cortisol to inactive cortisone	Josephson, et al. 2012
Receptor level	Systemic immune cells	ER22/23EK, GR isoform imbalance, aberrant GRα/GRβ	Impair GR transactivation and steroid sensitivity	Manenschijn, L., et al. 2009
	ILC2s	IL-13, IL-5	Disruption of the GRα/GRβ ratio	AbuJabal, et al. 2024
	AECs	TSLP, IL-33, IL-25	Activate ILC2s and disturb pre‑steroid airway homeostasis	Clancy, D.M., et al. 2018
	Epithelial/immune cells	IL-13	Block GR nuclear translocation	Gu, et al. 2024 Sahnoon, et al. 2025
	Immune cells	IL-6, TNF-α, defective GR chaperones, abnormal membrane GR signaling	Disrupt GR phosphorylation, DNA binding, nuclear trafficking, and transcriptional initiation	Manenschijn, et al. 2009 Russcher, et al. 2005
	B cells	IgG/IgA immune complexes, C3a/C5a	Perpetuate complement-driven neutrophilic inflammation to impair GR function	Ricklin, D., et al. 2007
Post-receptor	Epithelial/immune cells	Oxidative stress, cytokine signaling	Reduce HDAC2 activity/expression and impair GR transcriptional efficacy	Liu, et al. 2025
	Th17 cells	IL-1β/STAT5 axis	Alter GR transcriptional activity and repress anti‑inflammatory genes	Miller-Little, et al. 2024
	Neutrophils	Neutrophil cytoplasts, IL-1β	Promote Th17 metabolic reprogramming	Miller-Little, W.A., et al. 2024
	M1 macrophages	TNF-α, IL-1β, IL-6	Perpetuate pro-inflammatory signaling	Sharma, N., et al. 2018 Vargas, J.E., et al. 2016
	M1 macrophages	Sustained STAT3 activation	Reduce p‑GR and SOCS3 to block GR anti‑inflammatory signaling	Xue, et al. 2021

Abbreviations: AEC: arway epithelial cell; GR: glucocorticoid receptor; p‑GR: phosphorylated glucocorticoid receptor; TSLP: thymic stromal lymphopoietin; IL: interleukin; ILC2s: group 2 innate lymphoid cells; Th: T helper; TNF-α: tumor necrosis factor-alpha; HDAC2: histone deacetylase 2; STAT: signal transducer and activator of transcription; SOCS3: suppressor of cytokine signaling 3; Ig: immunoglobulin; C3a: Complement component 3a; C5a: Complement component 5a; 11β‑HSD2: 11β-hydroxysteroid dehydrogenase type 2; MDR1: multi-drug resistance type 1; IFN: interferon

### Therapeutic strategies targeting cellular crosstalk nodes in NA

Building upon the intricate cellular networks in NA, the consistent failure of single-target therapies calls for a paradigm shift from reductionist approaches to systems-level “network regulation”. Because steroid resistance arises from self-perpetuating multicellular interactions converging on GR dysfunction, identifying critical network hubs and designing rational combination therapies is a promising avenue to reverse resistance.

Frontline strategies aim to disrupt the neutrophil‑epithelial axis at multiple levels. One major focus is targeting NETs through three modalities: inhibiting formation, promoting degradation, and modulating release [64]. Specifically, formation can be inhibited by targeting peptidyl arginine deiminase 4 (e.g., **Cl-amidine**) [65], NE (e.g., **Sivelestat**) [66], or myeloperoxidase (e.g., **AZM198**) [67]; degradation enhanced by deoxyribonuclease I **(DNase I)** or macrophage clearance [68]; and release modulated by antioxidants (e.g., **N-acetylcysteine**) or C-X-C motif chemokine receptor 1/2 antagonists (e.g., **Reparixin**) [69, 70], thereby reducing pathological NETs while preserving beneficial functions. Beyond NETs, blocking epithelial alarmins offers complementary benefit. Monoclonal antibodies against TSLP (e.g., **Tezepelumab**), IL-33 (e.g., **Itepekimab**), or IL-25 interrupt downstream signaling after epithelial damage [71, 72]. Anti‑TSLP has shown efficacy in severe asthma, potentially by inhibiting ILC2-mediated IL-13/IL-5 production and reversing glucocorticoid resistance [73]. Notably, IL-33 promotes NETs formation via the mTOR-HIF-1α axis, creating a positive feedback loop; thus, anti-IL-33 may concurrently suppress neutrophil infiltration and their auto-amplification [74]. Additionally, targeting downstream ILC2 effector cytokines, anti-IL-13 and anti-IL-5, addresses sustained type 2 inflammation [75]. Anti‑IL‑5 (e.g., **Mepolizumab**) is effective in glucocorticoid‑resistant patients [23], and combining with anti-IL-13 (e.g., **Tralokinumab**) [76] could achieve more comprehensive blockade. Collectively, these multi-pronged strategies dismantle the pathological network at the neutrophil-epithelial interface by simultaneously targeting NETs, alarmins, and effector cytokines, aiming to resensitize the airway to corticosteroids (Table 2).

**Table 2:** Therapeutic agents targeting neutrophil-AEC interaction

Drugs	Utility	Preclinical/ Clinical Study	Etiology	Model	Dose	References
Cl-amidine	PAD4 inhibitor	Preclinical	Sepsis	Murine model	50 mg/kg	Biron, B.M., et al. 2017
Sivelestat	Neutrophil elastase inhibitor	Preclinical	LPS-ALI	Murine model	200 mg/kg	Fei, Y., et al. 2024
AZM198	MPO inhibitor	Preclinical	Cystic fibrosis lung disease	Murine model	125 µmol/kg	Dickerhof, N., et al. 2020
DNase I	NETs degradation agent	Preclinical	ALI	Rat model	100 U (≈ 3.33 mg/kg)	Du, Y., et al. 2023
Reparixin	CXCR1/2 inhibitor	Preclinical	Sepsis	Murine model	20 mg/kg	Alsabani, M., et al. 2022
N-acetylcysteine	Antioxidant and anti-inflammatory agent	Clinical	COPD	504 patients and 502 placebo-treated controls	600 mg twice daily	Zheng, J.P., et al. 2014
Tezepelumab	Anti-TSLP monoclonal antibody	Clinical	Asthma	529 patients and 532 placebo-treated controls	210 mg subcutaneously every 4 weeks	Menzies-Gow, A., et al. 2021
Itepekimab	Anti-IL-33 monoclonal antibody	Clinical	Asthma	74 received placebo, 73 received itepekimab, 74 received itepekimab +dupilumab and 74 received dupilumab	270 mg subcutaneously every 4 weeks	Corren, J., et al. 2021
Mepolizumab	Anti-IL-5 monoclonal antibody	Clinical	Asthma	385 Patients and 191 placebo-treated controls	75 mg intravenous or 100 mg subcutaneous every 4 weeks	Ortega, H.G., et al. 2014
Tralokinumab	Anti-IL-13 monoclonal antibody	Clinical	Asthma	70 patients and 70 placebo-treated controls	300 mg subcutaneously every 2 weeks	Busse, W.W., et al. 2019

Abbreviations: AEC: airway epithelial cell; PAD4: peptidyl arginine deiminase 4; LPS: lipopolysaccharide; ALI: acute lung injury; MPO: myeloperoxidase; NETs: neutrophil extracellular traps; CXCR: C-X-C chemokine receptor; COPD: chronic obstructive pulmonary disease; TSLP: thymic stromal lymphopoietin; IL: interleukin

Therapeutic approaches targeting the neutrophil-Th17 axis aim to correct its pathogenic imbalance and plasticity. Although IL-17 receptor blockade alone (e.g., **Brodalumab**) failed in clinical trials [11], a multi-level approach shows greater promise. This includes restoring T-bet to suppress Retinoic acid receptor-Related Orphan Receptor gamma t-driven Th17 differentiation, inhibiting steroid-induced metabolic reprogramming (e.g., STAT3/HIF-1α inhibitor **Stattic** to counter glucocorticoid-driven Th17 glycolysis and proliferation) [77, 78], and blocking cytokine-mediated resistance (e.g., employing IL-1 receptor antagonist **Anakinra** to disrupt the IL-1β/STAT5 axis) [33, 79, 80]. Collectively, this signifies a shift from single cytokine blockade to multi-level modulation of Th17 biology (Table 3).

**Table 3:** Therapeutic targeting of neutrophil crosstalk with other immune cells

Drugs	Utility	Preclinical/ Clinical Study	Etiology	Model	Dose	References
Stattic	JAK2/STAT3 inhibitor	Preclinical	LPS-induced AKI	Murine model	5 mg/kg or 10 mg/kg	Lee, S.-H., et al. 2024
Infliximab	TNF-α inhibitor	Preclinical	OVA-induced asthma	Murine model	5 mg/kg	Starkhammar, M., et al. 2015
MCC950	NLRP3 inflammasome inhibitor	Preclinical	NLRP3-driven inflammatory diseases	Cells	0.1–10 µM	Coll, R. C., et al. 2019
Brodalumab	Anti-IL-17Ra monoclonal antibody	Clinical	Asthma	228 patients and 77 placebo-treated controls	140/ 210/ 280 mg subcutaneously every 2 weeks	Busse, W.W., et al. 2013
Anakinra	Anti-IL-Ra antibody	Clinical	Pericarditis	224 patients	100 mg once daily by subcutaneous injection	Imazio, M., et al. 2020
Canakinumab	IL-1β inhibitor	Clinical	Autoinflammatory recurrent fever syndromes	456 patients	150 mg subcutaneously at the onset of a flare	Schlesinger, N., et al. 2012
Tocilizumab	Anti-IL-6Ra monoclonal antibody	Clinical	Early systemic sclerosis	104 patients and 106 placebo-treated controls	162 mg subcutaneously	Khanna, D., et al. 2022
N-acetylcysteine	Antioxidant and anti-inflammatory agent	Clinical	COPD	504 patients and 502 placebo-treated controls	600 mg twice daily	Zheng, J.P., et al. 2014
Eculizumab	Anti-C5 monoclonal antibody	Clinical	Hemolytic uremic syndrome	50 patients and 50 placebo-treated controls	300–1200 mg intravenously on days 0, 7, 14, 21, and 28.	Garnier, A., et al. 2023
Rituximab	Anti-CD20 monoclonal antibody	Clinical	nephrotic syndrome	78 patients	375 mg/m² intravenously once weekly for 4 weeks	Iijima, K., et al. 2022
Theophylline	phosphodiesterase inhibitor	Clinical	Asthma	50 patients and 50 placebo-treated controls	0.2 g twice daily	Wang, Y., et al. 2016
Vorinostat	Histone deacetylase inhibitor	Clinical	Cutaneous T-cell lymphoma	186 patients And 184 mogamulizumab treated controls	400 mg orally once daily	Kim, Y.H., et al. 2018

Abbreviations: JAK: janus kinase; STAT: signal transducer and activator of transcription; LPS: lipopolysaccharide; AKI: acute kidney injury; TNF-α: tumor necrosis factor α; OVA: ovalbumin; NLRP3: NOD-like receptor family, pyrin domain containing 3; IL: interleukin; COPD: chronic obstructive pulmonary disease; C5: complement component 5; CD20: cluster of differentiation 20

Strategies targeting neutrophil-macrophage interactions aim to dismantle the M1 polarization cycle that sustains steroid resistance. This requires a multi-tiered approach towards different nodes of the inflammatory cascade. At the effector cytokine level, anti-TNF-α (e.g., **Infliximab**) blocks NF-κB activation and reduces G-CSF‑driven neutrophil mobilization [43, 81]; anti-IL-1β (e.g., **Canakinumab**) alleviates neutrophilic airway hyperresponsiveness by inhibiting NLRP3 inflammasome activation [82]; and anti-IL-6 receptor (**Tocilizumab**) restores GR function via inhibiting STAT3‑mediated downregulation of GR phosphorylation and HDAC2 [44, 83, 84]. Upstream, direct NLRP3 inhibitors (e.g., **MCC950**) have shown promise in preclinical models [85]. Adjuvant antioxidants such as **N-acetylcysteine** can scavenge neutrophil-derived ROS to reduce M1-polarizing signals, with their efficacy likely being optimized when combined with the aforementioned cytokine-targeted biologics [69, 86]. This integrated, multi‑pronged strategy aims to decouple macrophage‑driven inflammation from neutrophil recruitment and resensitize the airway to corticosteroids (Table 3).

Therapies targeting the neutrophil-B cel autoimmune axis aim to break the autoantibody-complement‑neutrophil cycle. A foundational approach involves intercepting the downstream effector signals: complement inhibitors (e.g., anti-C5a antibody **Eculizumab**) block anaphylatoxin-mediated neutrophil chemotaxis [87]. Moving upstream, B cell-targeted therapies offer more direct intervention. Blocking B-cell activating factor, a critical regulator of B cell survival and antibody production, may reduce pathogenic autoantibody synthesis, a strategy particularly relevant given the association between IgA^+^ memory B cells and small airway dysfunction [53, 88]. For patients with a pronounced autoimmune phenotype, B cell depletion with anti-CD20 monoclonal antibodies (e.g., **Rituximab**) is an option, though immunosuppressive risks require caution [89]. Collectively, this multi-target approach aims to disrupt the cycle central to steroid resistance (Table 3).

A cornerstone strategy for overcoming steroid resistance involves direct targeting of GR dysfunction that lies at the convergence of all pathological networks. This approach aims to reset the core GR signaling apparatus to resensitize the inflammatory microenvironment. Two distinct but complementary sub‑strategies can be pursued. First, pharmacological enhancement of GR activity, exemplified by low-dose **Theophylline** as an HDAC2 activator that restores GR-mediated transrepression [90, 91]. Second, epigenetic modulation. It is important to note that the proposed use of an HDAC inhibitor is not mechanistically contradictory to HDAC2 activation; rather, it reflects a distinct therapeutic rationale. Low-dose Theophylline is designed to specifically activate the deficient HDAC2 isoform. In contrast, the pan‑HDAC inhibitor **Vorinostat** (targeting HDAC1, 2, 3, and 6) is predicated on broader epigenetic reprogramming. Its pleiotropic effects, including inhibition of other pro‑inflammatory HDACs and modulation of non‑histone protein acetylation, may reverse a pervasive pro‑inflammatory chromatin state and indirectly restore GR signaling [92]. Thus, Vorinostat could reinstate GR-dependent anti-inflammatory gene expression [93]. Together, these interventions, including restoring GR function and remodeling the epigenetic landscape, collectively aim to resensitize refractory NA by re-establishing GR as the central regulatory axis (Table 3).

### Integrating network-based therapeutics with patient stratification

Steroid resistance in NA is not a static molecular defect, but as a dynamic, self-perpetuating state sustained by a pathological network of neutrophils, AECs, T cells, macrophages, and B cells. This network’s resilience and redundancy may explain the consistent failure of single-target therapies (e.g., anti-IL-17), as compensatory pathways rapidly restore inflammatory equilibrium. Overcoming this requires a shift from single-target inhibition to multi-target network disruption, with rationally designed combination therapies that simultaneously target critical hubs. For example, suppressing neutrophil activation, disrupting neutrophil‑Th17 crosstalk, and neutralizing GR-impairing mediators from M1 macrophages could compromise the network’s integrity, reset the inflammatory microenvironment, and restore GR function. Ultimately, this “network‑therapeutics” approach reframes the goal from silencing a single target to resetting an entire pathological system. Translating this framework into clinical practice will require patient‑specific biomarker profiling to map dominant pathogenic circuits, enabling personalized combination therapies that restore durable immune homeostasis, a potential advance in NA management.

The profound heterogeneity in treatment responses among NA patients calls for a shift from broad phenotyping to precision medicine, guided by discriminative biomarkers that reflect dominant pathological circuits. We propose an integrative endotyping framework that stratifies patients based on actionable biomarker signatures, aligning them with mechanism targeted therapies. The framework delineates five putative endotypes: (1) the epithelial-neutrophil axis-dominated endotype, characterized by elevated NETs components, NE, and alarmins (e.g., TSLP, IL-33), implicating a vicious cycle that could be interrupted by targeting both neutrophil recruitment (e.g., C-X-C motif chemokine receptor 1/2 antagonists Reparixin) and alarmin signaling (e.g., anti-IL-33 Itepekimab); (2) the Th17-neutrophil circuit endotype, defined by high levels of IL-17 A/F, IL-22, and neutrophil cytoplasts, suggesting a self-amplifying loop potentially underpinned by T-bet dysregulation; (3) the macrophage-polarization dominant endotype, accompanied by elevated M1-associated cytokines (TNF-α, IL-1β, IL-6), implying a therapeutic approach combining NLRP3 inflammasome inhibitors (e.g., MCC950) with antioxidants (e.g., N-acetylcysteine) to reduce IL-1β maturation and ROS-driven M1 polarization; (4) the B cell/autoimmune-like endotype, marked by increased IgG/IgA, autoantibodies, complement fragments, and IgA^+^ memory B cells, revealing an immune complex/complement-driven pathway distinct from type 2 inflammation; and (5) a mixed phenotype, combining features of multiple endotypes, likely representing a more complex and treatment-resistant variant requiring multi-pronged strategies. This biomarker‑guided stratification is intended as the cornerstone for deploying rational combination therapies, enabling targeted disruption of a patient’s predominant dysregulated network.

To render this endotyping framework actionable, specific measurable biomarkers must be nominated together with sample types, assay methods, and preliminary thresholds. Based on the network axes described above, we propose three priority candidates. The sputum IL‑1β/IL‑8 ratio reflects neutrophil‑macrophage crosstalk, with an elevated ratio suggesting a macrophage‑driven resistance circuit (measured by multiplex ELISA or Luminex in induced sputum supernatant) [94]. NE activity or citrullinated histone H3 (Cit-H3) serves as a NET‑specific functional marker. Sputum NE activity > 20 µg/mL or Cit-H3 > 11.25–22.58 ng/mL depending on the studies [95, 96] provisionally identifies patients with high NET burden (NE activity is measured by fluorometric assay, and Cit‑H3 by ELISA). Sputum IL‑33 (measured by high‑sensitivity ELISA) at concentrations > 18–77 pg/mL [97] has been associated with corticosteroid resistance. Induced sputum is the preferred sample type, with blood as backup; bronchoalveolar lavage is too invasive for routine use. To enable prospective validation, we propose a multicenter, prospective observational cohort study in adults with severe asthma, sputum neutrophils ≥ 40%, and documented glucocorticoid resistance. The study aims to test whether the nominated candidate biomarkers can independently predict future exacerbation risk or glucocorticoid resistance in patients with NA. The primary endpoint would be the annualized rate of asthma exacerbations over a period of 52 weeks. Secondary endpoints include the forced expiratory volume in 1 s, scores on the Asthma Control Questionnaire-6, and Asthma Quality of Life Questionnaire. Sample size will be calculated to ensure sufficient statistical power based on anticipated medium effect sizes for the primary biomarker candidates. Receiver operating characteristic analysis will be used to determine optimal thresholds, and multivariable regression will adjust for confounders. We acknowledge that the proposed threshold values are currently hypothetical and require prospective validation; assays for NE activity and Cit-H3 also need technical standardization. A cross‑center reproducibility substudy is recommended before clinical trial application. Success in this endeavor would advance a truly precision medicine approach for severe, steroid-resistant asthma, transforming a complex pathological understanding into an actionable diagnostic and therapeutic strategy.

## Conclusions and future perspectives

This review advances the paradigm that glucocorticoid resistance in NA is orchestrated by a resilient, dysregulated cellular network rather than by isolated defects (Fig. 1). While this integrated framework provides a coherent pathophysiological model and uncovers novel therapeutic nodes, its development and translation are confronted by several layers of uncertainty. A primary limitation stems from the nature of the available evidence, which is largely derived from reductionist preclinical models and cross-sectional human studies. These approaches, though invaluable for deconstructing specific binary interactions (e.g., neutrophil-epithelium), inherently fail to recapitulate the dynamic, multi-cellular crosstalk occurring concurrently in human disease. Consequently, the hierarchy, temporal dynamics, and reciprocal regulation among different network axes (e.g., neutrophil-macrophage *versus* B-cell-complement) remain poorly defined. This lack of temporal resolution is more than a theoretical concern: without knowing which axis acts as the dominant “master regulator” over time, it is exceedingly difficult to determine exactly when and where to safely intervene with combination therapies. Based on the available evidence, we propose a putative temporal model for NA pathogenesis. The neutrophil-epithelial axis likely serves as the initiating hub; epithelial damage and alarmin release are early events that recruit and activate neutrophils. This is followed by a parallel amplification phase involving neutrophil-Th17 and neutrophil-macrophage crosstalk, which sustains and exaggerates inflammation. The B-cell-complement axis probably represents a later, self-perpetuating loop that emerges after repeated tissue injury and antigen exposure, driving chronicity and steroid resistance. However, this hierarchy remains hypothetical and requires direct validation. We therefore outline an experimental roadmap: (1) longitudinal sampling of airway specimens (sputum, bronchoalveolar lavage, biopsies) from newly diagnosed NA patients to map the temporal appearance of key mediators; (2) use of human airway organoid-immune cell co-culture systems to perturb individual axes and measure cross-activation kinetics; and (3) spatial transcriptomics of archived tissue to infer cellular interaction timelines based on proximity and activation states.

Translating this network paradigm into clinical practice faces parallel challenges. The biomarker signatures central to the proposed endotypic stratification, such as specific NETs components or T cell cytoplasts, currently lack standardized, clinically validated assays. Most critically, the promising rationale for network-informed combination therapy remains a theoretical construct, and multi‑target therapy carries substantial risks of adverse reactions. To bridge this gap, we propose two converging strategies. First, advanced preclinical validation: candidate regimens should be tested in human airway organoid-immune cell co-cultures and lung-on-a-chip systems. These platforms can model sequential versus simultaneous axis inhibition (e.g., NETosis plus anti-IL-1β) and quantify off-target immunosuppression. Only combinations that achieve synergistic network disruption without global immune paralysis should advance. Second, adaptive and modular trial designs: traditional fixed-dose combination trials are ill-suited for a dynamic network. Instead, biomarker-guided adaptive designs (platform trials, sequential multiple assignment randomized trials) are preferable. Patients would start with a single agent targeting their dominant endotype; if resistance emerges or the axis shifts, additional agents are added sequentially under real-time molecular monitoring. Thus, combination therapy need not mean “all agents from day one”, staggered, response-adaptive modulation is a safer near-term goal. The proposed combination regimens and their current level of evidence are summarized in Table 4. Advances in artificial intelligence and nanomedicine hold promise to further tackle this dilemma. Artificial intelligence has already been applied to identify and design multi-target ligands for asthma [98], and nanomedicine could enable local delivery of combination agents (e.g., inhaled nanoparticle‑encapsulated biologics), reducing systemic immunosuppression. Furthermore, high‑throughput microarrays and other monitoring technologies can enhance analytical precision for deciphering disease temporal dynamics and multicellular signal crosstalk [99].

**Table 4:** Proposed combination therapeutic strategies for NA

Proposed Combination	Individual Component Evidence	Combination Evidence
NETs inhibitor (e.g., Cl-amidine) + anti-TSLP (Tezepelumab)	Cl-amidine: murine sepsis model [65]; Tezepelumab: Phase III trial in asthma [72]	None published
NETs inhibitor + anti-IL-33 (Itepekimab)	Itepekimab: Phase II asthma trial [71]; NETs-IL-33 axis in murine smoke-exposure asthma [16]	None published
NE inhibitor (Sivelestat) + anti-IL-1β (Canakinumab)	Sivelestat: murine ALI model [66]; Canakinumab: Phase III trial in gout/autoinflammatory diseases [82]	None published
Anti-IL-17 (Brodalumab) + anti-TNF-α (Infliximab)	Brodalumab: Phase IIb trial in asthma (failed as monotherapy) [11]; Infliximab: murine asthma model [81].	None in asthma; tested in psoriasis [100]

Abbreviations: ALI: acute lung injury; NETs: neutrophil extracellular traps; NE: neutrophil elastase; TNF-α: tumor necrosis factor-alpha; TSLP: thymic stromal lymphopoietin; IL: interleukin

In summary, this review establishes a “cellular network” paradigm for glucocorticoid resistance in NA, providing a transformative framework for future research. The immediate priority is to decipher this network’s architecture using advanced technologies: organoid co-culture systems to model multicellular interactions; single-cell and spatial transcriptomics to define cellular states and map the tissue topography of crosstalk [101–103]. Integrating these tools will generate a high-resolution atlas of the disease, identifying novel therapeutic nodes. Emerging multi-target therapeutic strategies for NA integrate systems biology and bioinformatics-based multi-omics analysis to delineate intrinsic cellular crosstalk and temporal dynamics [104, 105], with nanomedicine supporting clinical translation. Translationally, this paradigm necessitates a shift toward precision medicine trials. Future studies must stratify patients by specific biomarker-defined endotypes (e.g., the epithelial-neutrophil axis) to test rational combination therapies (e.g., NETs inhibitors combined with anti-alarmin biologics). This “biomarker-guided network targeting” is essential for validating the framework’s clinical utility. Strikingly, the Th17/neutrophil‑centric network described here shows conceptual similarities to pathologies in conditions such as psoriasis vulgaris, which also features dysregulated Th1/Th17 axes [106, 107]. This observation is speculative and hypothesis‑generating rather than evidence‑based. Currently, no systematic comparative data exist on cellular interactions, cytokine profiles, or steroid responsiveness across NA, psoriasis, and rheumatoid arthritis. Whether a conserved “core immune network dysregulation” underlies these diseases remains an open question that requires direct investigation. Future studies could perform comparative analyses of shared pathogenic circuits, for example, by evaluating NETosis, IL‑17 signaling, and steroid sensitivity in parallel across disease cohorts. Testing such network‑based hypotheses will require the integration of advanced technologies and cross‑disciplinary frameworks. Until such evidence emerges, any extrapolation of therapeutic mechanisms (e.g., anti‑IL‑17 efficacy in psoriasis to NA) should be considered tentative and hypothesis‑generating.

## Data Availability

The data and materials are available from the corresponding author on reasonable request.
